# Tryptophan-Based Hyperproduction of Bioindigo by Combinatorial Overexpression of Two Different Tryptophan Transporters

**DOI:** 10.4014/jmb.2308.08039

**Published:** 2023-11-30

**Authors:** Hyun Jin Kim, Sion Ham, Nara Shin, Jeong Hyeon Hwang, Suk Jin Oh, Tae-Rim Choi, Jeong Chan Joo, Shashi Kant Bhatia, Yung-Hun Yang

**Affiliations:** 1 Department of Biological Engineering, College of Engineering, Konkuk University, Seoul 05029, Republic of Korea; 2 Department of Chemical Engineering, Kyung Hee University, Yongin-si, Gyeonggi-do 17104, Republic of Korea; 3 Institute for Ubiquitous Information Technology and Application, Konkuk University, Seoul 05029, Republic of Korea

**Keywords:** Bioindigo, tryptophan transporter, *aroP*, *tnaB*, flavin-containing monooxygenase, tryptophanase

## Abstract

Indigo is a valuable, natural blue dye that has been used for centuries in the textile industry. The large-scale commercial production of indigo relies on its extraction from plants and chemical synthesis. Studies are being conducted to develop methods for environment-friendly and sustainable production of indigo using genetically engineered microbes. Here, to enhance the yield of bioindigo from an *E. coli* whole-cell system containing tryptophanase (TnaA) and flavin-containing monooxygenase (FMO), we evaluated tryptophan transporters to improve the transport of aromatic compounds, such as indole and tryptophan, which are not easily soluble and passable through cell walls. Among the three transporters, Mtr, AroP, and TnaB, AroP enhanced indigo production the most. The combination of each transporter with AroP was also evaluated, and the combination of AroP and TnaB showed the best performance compared to the single transporters and two transporters. Bioindigo production was then optimized by examining the culture medium, temperature, isopropyl β-D-1-thiogalactopyranoside concentration, shaking speed (rpm), and pH. The novel strain containing *aroP* and *tnaB* plasmid with *tnaA* and *FMO* produced 8.77 mM (2.3 g/l) of bioindigo after 66 h of culture. The produced bioindigo was further recovered using a simple method and used as a watercolor dye, showing good mixing with other colors and color retention for a relatively long time. This study presents an effective strategy for enhancing indigo production using a combination of transporters.

## Introduction

For centuries, the distinctively colored blue dye indigo has been used worldwide in the textiles, art, and cosmetics industries [1-3]. However, as the traditional method of extracting indigo from plants is time-consuming and requires large amounts of land for cultivation [4, 5] a chemical synthesis method for producing indigo has been developed. This chemical synthesis of indigo, however, poses a serious threat to waterbodies due to the toxic chemicals and reducing agents that include formaldehyde, hydrogen cyanide, sodium amide, and strong bases used in the process [6, 7]. Therefore, research has been conducted to produce indigo using biotechnological approaches as an alternative to chemical synthesis [8].

The microbial production of indigo is eco-friendly, with low environmental impact, and requires only mild reaction conditions [9]. In this study, bioindigo means indigo synthesized from recombinant microorganisms. Previous studies have reported bioindigo production in microorganisms such as *Pseudomonas putida*, *Acinetobacter*, *Comamonas*, and *Escherichia coli* [10-14]. These studies reported that the microbes used indole, phenol, and tryptophan as substrates for the biosynthesis of indigo and that oxygenase enzymes, such as styrene monooxygenase, naphthalene dioxygenase, and flavin-containing monooxygenase, played significant roles in this process. In particular, many studies have been conducted to produce bioindigo from indole using flavin-containing monooxygenase (FMO) in *E. coli*. This relies on NADPH to oxidize nucleophilic N- and S-containing compounds and is found in microorganisms and plants, such as *Corynebacterium glutamicum*, *Nitrincola lacisaponensis*, *Methylophaga aminisulfidoran*, and *Polygonum tinctorium* [15]. However, indole is toxic to cells and cannot be used as a substrate at concentrations greater than 3 mM, limiting the large-scale production of bioindigo [16]. Therefore, studies using tryptophan as an alternate substrate for mass production of bioindigo have recently been reported (Table 1). Tryptophan is converted to indole by tryptophanase (*tnaA*), and *E. coli* has the *tnaA* gene [17]. Indole is converted to indoxyl by an oxygenase, which can be dimerized in the presence of oxygen to produce indigo [18]. In our previous study, we developed a bioindigo production system in *E. coli* by introducing tryptophanase (*tnaA*) derived from *E. coli* K12 and *FMO* derived from *M. aminisulfidivorans* [19].

In microorganisms, the transport process plays crucial roles in cellular metabolism, absorption of nutrients, excretion of waste products, and maintenance of intracellular homeostasis [20]. Amino acid absorption by microorganisms can be controlled by introducing amino acid transporter genes. This can be applied in many practical ways [21]. For example, the deletion of *brnQ*, a branched-chain amino acid permease, reduces the uptake of L-isoleucine in *C. glutamicum*, resulting in the production of 170.3 mM (22.3 g/l) of L-isoleucine [22]. This study is focused on regulating tryptophan absorption and utilization in *E. coli* by introducing tryptophan transporter genes. In previous studies, the introduction of *tnaB*, an aromatic amino acid permease, increased the absorption of tryptophan by *C. glutamicum*, resulting in the production of 0.9 g/l of indole [23]. In another study, the deletion of *tnaB* also reduced indole production by 20% in *E. coli* [24]. Increased tryptophan intake can have downstream effects on various metabolic pathways that depend on tryptophan as a precursor, including the production of bioactive compounds or secondary metabolites.

In this study, to enhance the production of bioindigo with an *E. coli* whole-cell system containing tryptophanase (TnaA) and flavin-containing monooxygenase (FMO), we evaluated tryptophan transporters to improve the transport of aromatic compounds, such as indole and tryptophan, which are not easily soluble and passable through cell walls. In addition, the synergy effect of simultaneous expression of the tryptophan transporter was compared, and the effect of the introduction of the transporter on the substrate and intermediate products was confirmed. This study presents an effective strategy that uses microorganisms to enhance indigo production by overexpressing a combination of transporters.

## Materials and Methods

### Chemicals

Tryptophan and indigo were purchased from Sigma-Aldrich (USA). Indole was obtained from Alfa Aesar (USA). Dimethyl sulfoxide (DMSO) was purchased from Samchun Chemicals (Republic of Korea). Isopropyl-β-D-thiogalactopyranoside (IPTG) (>99%) was obtained from Biosesang Co., (Republic of Korea). All other media components were purchased from Difco (USA).

### Plasmid Construction and Strains

Information on the bacterial strains and plasmids used in this study is presented in (Table 2). E coli DH5α and *E. coli* BL21(DE3) were used as hosts for genetic recombination and indigo production, respectively [25]. For tryptophan metabolism, a pCDFDuet-1 vector carrying the tryptophanase gene (*tnaA*) and the flavin-containing monooxygenase gene (FMO) was used [26]. For the tryptophan and indole transport systems, the tryptophan transporters *aroP*, *tnaB*, and *Mtr* were inserted into a pET 24ma vector. The genes were inserted into plasmids by T4 cloning, followed by transformation into *E. coli* DH5α by heat shock.

### Media and Culture Conditions

Cells were pre-cultured in lysogeny broth (LB) containing 10 g/l tryptone, 5 g/l yeast extract, and 5 g/l sodium chloride at 37°C [27]. Pre-culture was performed by inoculating a single colony of *E. coli* BL21 (DE3) picked from LB agar plates into 5 ml of LB medium. The culture medium was incubated at 37°C for 18 h with agitation at 200 rpm. To produce indigo, the 18 h pre-culture was inoculated at a dilution of 1:50 (v/v) into 5 ml of Terrific broth (TB, casein 12 g/l, yeast extract 24 g/l, dipotassium phosphate 9.4 g/l, monopotassium phosphate 2.2 g/l) containing tryptophan 15 mM. Appropriate concentrations of antibiotics (100 μg/ml spectinomycin and 50 μg/ml kanamycin) were used to ensure plasmid incorporation. After approximately 2 h of incubation at 37°C, the OD_600_ reached 0.8, and the culture was then induced by the addition of 0.01 mM of isopropyl-beta-D-thiogalactoside (IPTG). The culture was further incubated at 25°C for 48 to 72 h.

### Determination of Bioindigo

To analyze indigo production, culture samples were centrifuged at 13,000 ×*g* for 15 min, washed twice with deionized water, and resuspended in 1 ml DMSO. The suspended samples were homogenized using BeadBug 6 homogenizer (Benchmark Scientific, USA) and centrifuged at 5,500 ×*g* for 90 s. The resulting supernatant was diluted to the appropriate concentration with DMSO and transferred to 96-well plates; the indigo concentration was estimated by reading the absorbance at 620 nm using a SpectraMax M2 UV-Vis spectrophotometer (Molecular Devices, USA) [28].

### Determination of Tryptophan and Indole

Tryptophan and indole concentrations were quantified using an HPLC equipped with a UV/Vis Detector (YL-9100, Republic of Korea) [29]. Samples were detected at 280 nm using a reverse-phase C18 column (ZORBAX SB-C18 column, 4.6 × 250 mm, 5 μm particle size, Agilent Technologies, USA). The oven temperature was maintained at a constant 35°C during operation. Water and 100% methanol were used as mobile phases A and B, respectively. The flow rate was maintained at 0.8 ml/min using the following gradient program: (A: B, v/v) 0–20 min; (80:20) 20–25 min; (20:80) 25–34 min; (20:80) 34–38 min; (80:20) and 38–40 min; (80:20). For sample preparation, the supernatant was filtered through a 0.22-μm Millex-GP syringe filter unit and diluted with HPLC-grade water. The filtered samples were diluted and transferred to vials for the detection of tryptophan and indole. The retention time of each sample was 10.5 min and 30.4 min, respectively.

### Bioindigo Recovery Method

The bioindigo in the cells was recovered using a 1% SDS. The *aroP*+*tnaB* strain was cultured in a 100-ml flask under the optimized conditions of the previous experiment. After 66 h of incubation, detergent was added at a concentration of 10 to 20% of the total volume of the culture medium. The culture medium and the detergent were autoclaved to destroy the cells. After centrifugation, samples were washed twice with distilled water. To obtain bioindigo in the form of dry powder, the cells were dried in a 70°C dry oven for 30 min. The suitability of bioindigo was confirmed by dissolving the dried indigo powder in water-colored paint; the bioindigo was mixed with other different colors.

## Results and Discussion

### Enhancement of Indigo Production by the Introduction of a Transporter

**Fig. 1:**
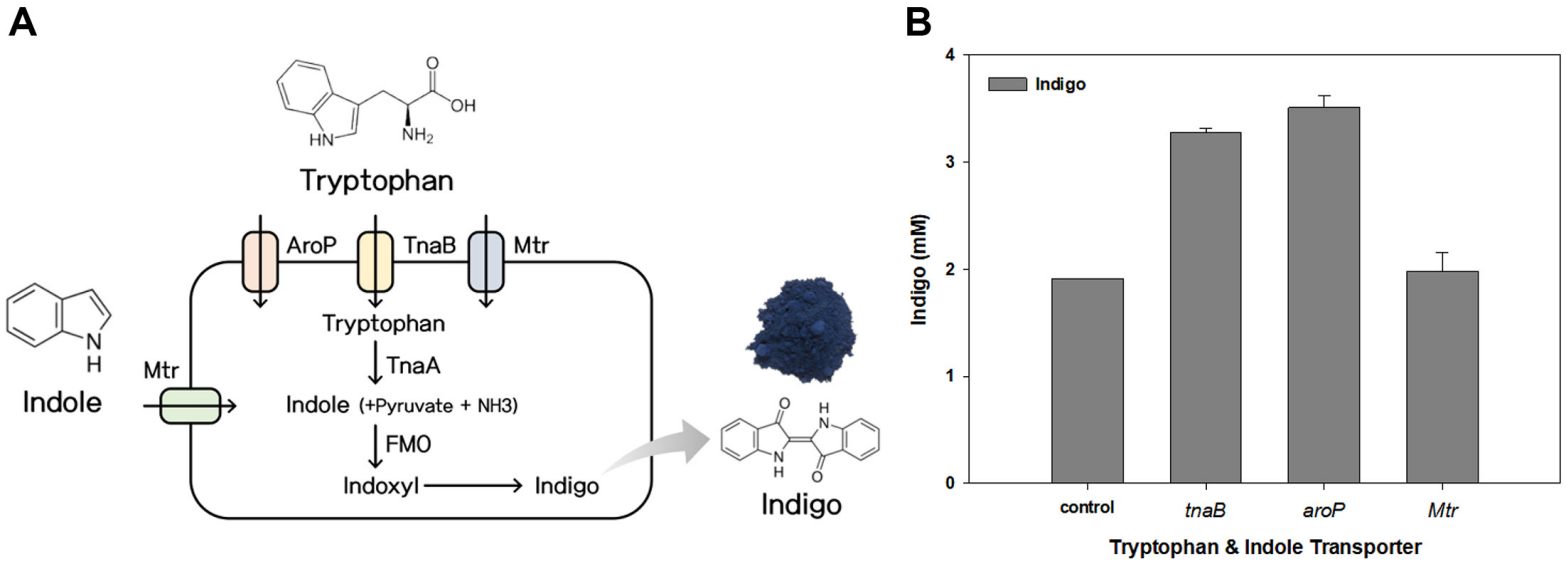
Indigo production of tryptophan and indole transporter genes in *Escherichia coli* BL21. (**A**) Metabolic pathway. (**B**) Effects of transporter gene on bio-indigo production.

The substrates used for indigo production by microorganisms include tryptophan and indole [30]. According to previous studies, a high titer of bioindigo was achieved by introducing the *FMO* gene from *M. aminisulfidivorans* and the *tnaA* gene [31]. In a well-known pathway, tryptophan is converted to indole by the expression of *tnaA* in the host strain, and then indole is converted to indigo by the expression of the oxygenase gene. Due to the chemical structure of indole and tryptophan, their transport is not easy. In addition to the toxicity of molecules and the low solubility of substrates, the transport of substrates is responsible for relatively low bioindigo production [32, 33]. In *E. coli*, three permeases, *aroP*, *tnaB*, and *Mtr*, are involved in the uptake and accumulation of L-tryptophan [24]. To increase the transport of tryptophan into the cell, the *aroP*, *tnaB*, and *Mtr* genes were introduced into the host cell *E. coli* BL21 (Fig. 1A). *AroP* is a general aromatic amino acid permease that transports tryptophan, phenylalanine, and tyrosine with high affinity [34-36]. *TnaB* is a low-affinity transporter specific to L-tryptophan [37, 38]. *Mtr* is a high-affinity transporter enzyme specific for L-tryptophan and can also transport indole [39, 40]. Studies have reported an increase in tryptophan and indole production when the tryptophan uptake was regulated via the overexpression or deletion of *aroP*, *tnaB*, and *Mtr* [41, 42]. However, no studies have been conducted on indigo production using transporter genes in microorganisms.

After constructing each transporter in the *FMO*-*tnaA* strain, bioindigo production was compared as described in the Materials and Methods section. Compared to the control expressing only *FMO* and *tnaA*, more indigo production was observed when the transporter genes were expressed together (Fig. 1B). The highest production was found in strains with the *aroP* gene, followed by strains with the *tnaB* gene.

**Fig. 2:**
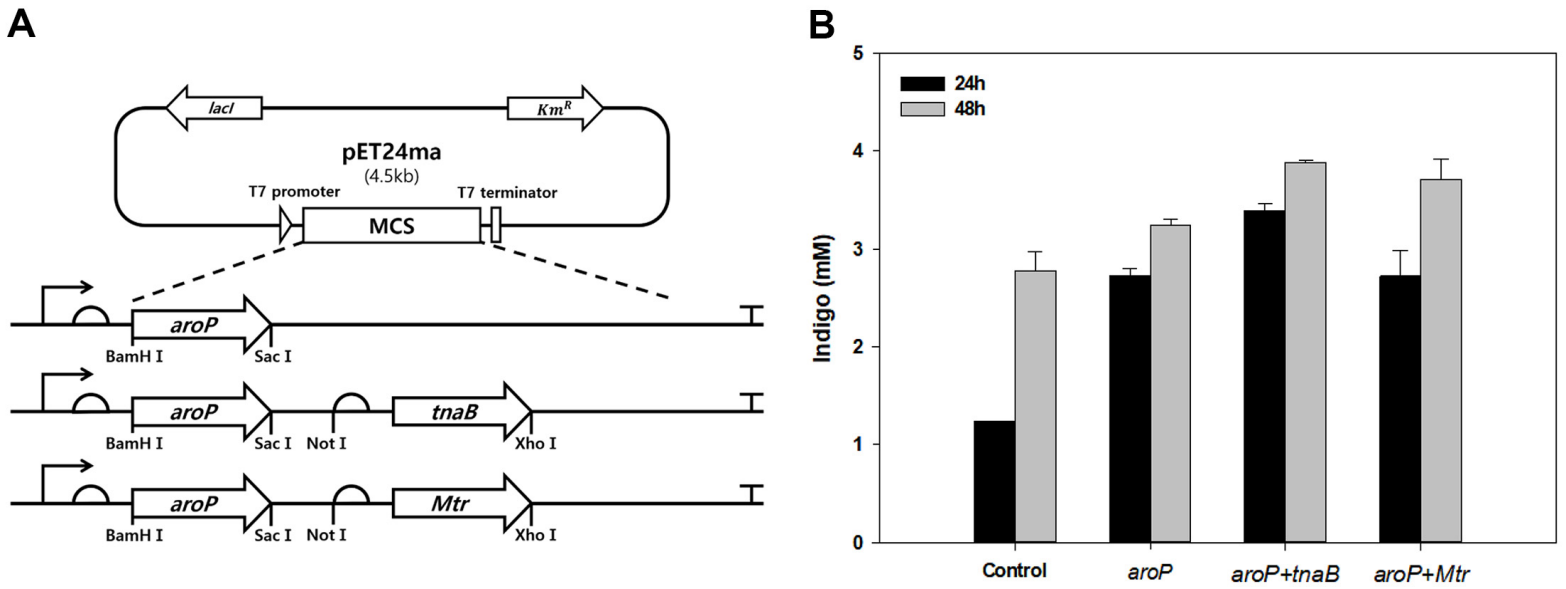
Screening of transporter genes for indigo production. (**A**) Introduction of transporter genes from *E. coli* K12. (**B**) Simultaneous expression of tryptophan and indole transporter genes including *aroP* gene.

To examine the synergistic effect of the transporters, we constructed recombinant *E. coli* BL21 that simultaneously expressed *aroP* and other transporter genes (Fig. 2A). As a control group, *E. coli* without the transporter gene and recombinant *E. coli* expressing only *aroP* gene were used. When tryptophan was used as a substrate, the synergistic effect of the simultaneous expression of *aroP* and other transporter genes was confirmed in both 24 h and 48 h cultures. Among them, the *aroP*+*tnaB* strain was the most efficient, producing indigo at a concentration of 3.87 mM at 48 h (Fig. 2B). In this study, a pCDFduet-1 vector containing *tnaA* and *FMO* genes was used as an indigo-producing plasmid. In addition, a pET24ma vector containing *aroP* and *tnaB* genes was used as the tryptophan transporter plasmid for subsequent experiments.

### Comparison of Indole Inside and Outside of Cells in Wild-Type and Engineered Strains

Tryptophan permeases *aroP* and *tnaB* are involved in the absorption of L-tryptophan in *E. coli* [43]. Previous studies have shown that these permeases function in metabolism and cellular transport, influencing the intracellular accumulation and secretion of metabolites [44] Therefore, the production of indole, a tryptophan metabolic intermediate that accumulates inside and outside cells, was confirmed in the *aroP*+*tnaB* strains. *E. coli* harboring indigo-producing plasmids only was used as a control. The culture was performed at 6 h intervals for 48 h with 40 mM tryptophan added to the culture medium.

**Fig. 3:**
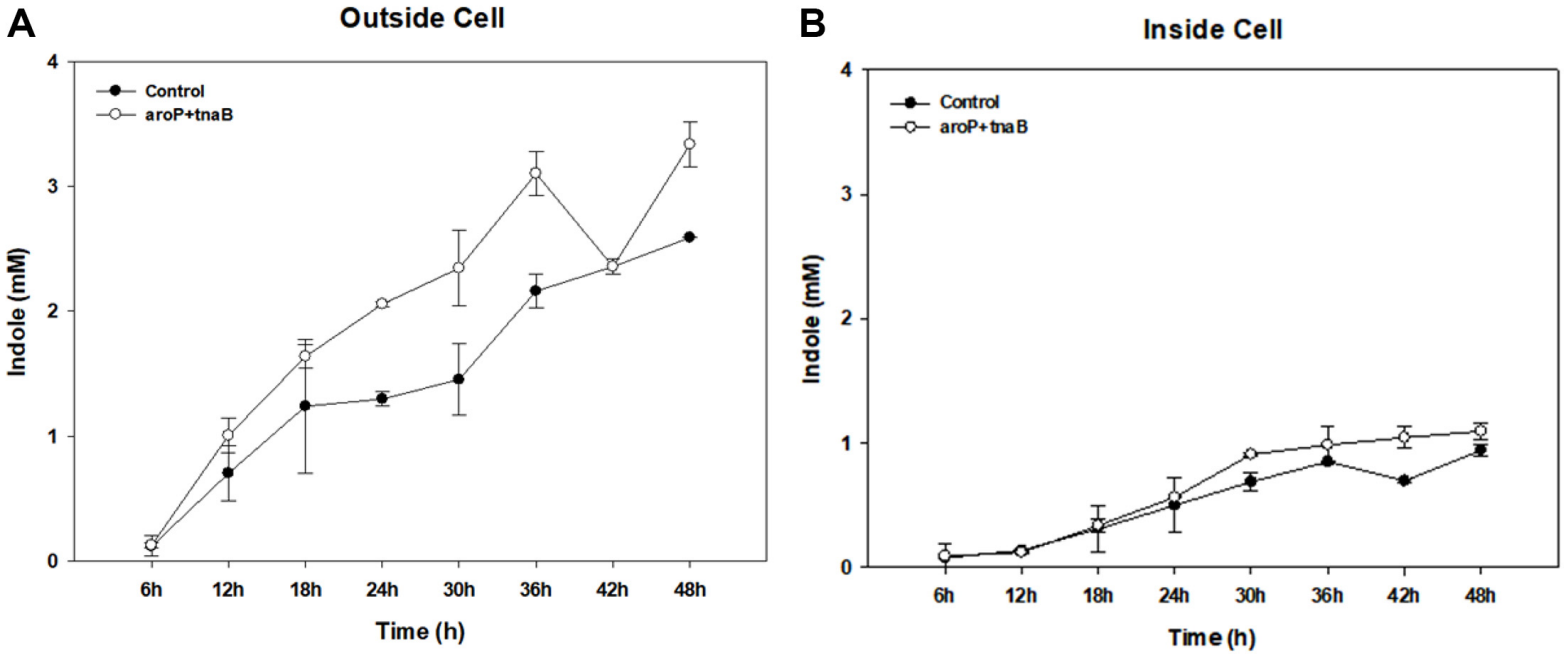
Comparison of indole inside and outside of cells in wild-type and engineered strains. (**A**) Outside cell. (**B**) Inside cell.

Indole accumulation increased over time, both outside and inside the cell, and more indole accumulation was observed in the *aroP*+*tnaB* strains. The accumulation of indole for 48 h outside the cell was 3.34 mM in the *aroP*+*tnaB* strain and 2.59 mM in the control, indicating 1.3 times higher production by the recombinant strains (Fig. 3A). Inside the cell, the *aroP*+*tnaB* strain showed 1.1 times higher production than the control group with the *aroP*+*tnaB* strain producing 1.1 mM, and the control group producing 0.94 mM (Fig. 3B). Introduction of the transporters *aroP* and *tnaB* increased the uptake of tryptophan by *E. coli*, suggesting that indole, an intermediate product of tryptophan metabolism, accumulates as the amount of metabolism increases.

When tryptophan inside and outside was measured, the overall amount of tryptophan in the *aroP*+*tnaB* strain was lower than the strain without transporters, suggesting that tryptophan was significantly consumed by *aroP*+*tnaB* strain to produce bioindigo, and that the introduction of transporters increased the utilization of tryptophan (data not shown). Overall, although it was not easy to directly introduce indole and tryptophan, the introduction of *aroP* and *tnaB* clearly changed the amount and utilization of both indole and tryptophan, resulting in different levels of bioindigo production.

### Optimization of Reaction Parameters for Bioindigo Production

**Fig. 4:**
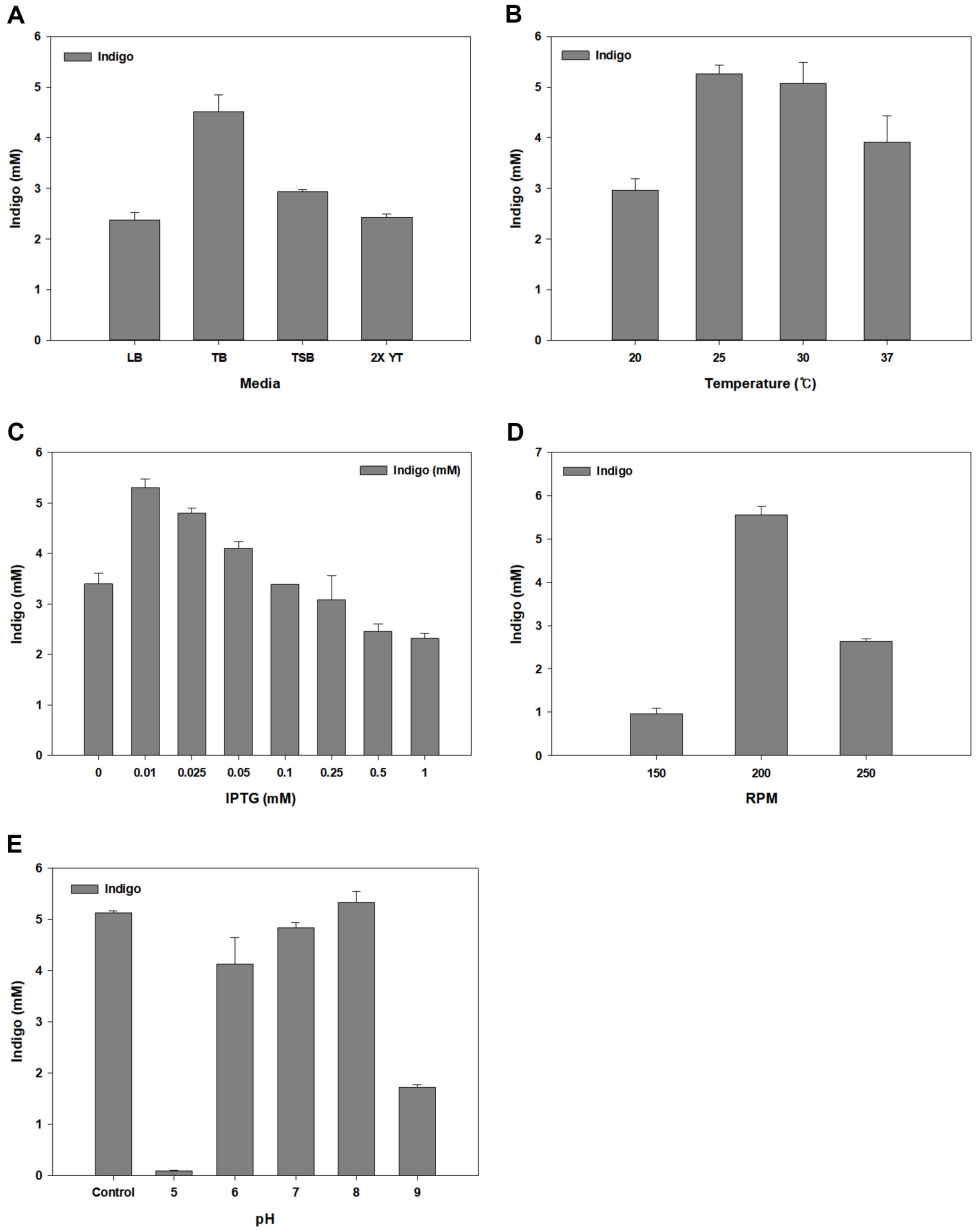
Optimization of culture condition. Indigo absorbance at 620 nm of the *aroP* and *tnaB* co-expression strain by (**A**) culture medium (**B**) temperature (**C**) IPTG concentration (**D**) RPM (**E**) pH.

Various culture optimization experiments with *aroP*+*tnaB* strain were conducted. Several culture conditions and media compositions were optimized for the mass production of bioindigo. All optimization experiments were performed in media supplemented with 15 mM tryptophan. First, we performed medium optimization. LB, TSB, TB, and 2X YT media were used as the culture media. Equal amounts of precultured cells (OD_600_ = 0.8) were added to the culture medium and incubated for 48 h to compare bioindigo production (Fig. 4A). The bioindigo production was 4.5 mM in TB medium, which was approximately twice as high as that in LB medium. This indicates that TB medium is the most suitable culture medium for indigo production, and TB media was used as the culture medium in subsequent experiments.

The culture temperature was then optimized. Depending on the temperature, the tertiary structure of the protein affects protein denaturation, off-folding, and enzyme activity [45]. This optimization process involved determining the optimal induction temperature at which the protein was actively expressed and maximum production of bioindigo occurred. Strain *aroP*+*tnaB* was cultured at 20, 25, 30, and 37°C after IPTG induction. The highest concentration (5.27 mM) was produced when the culture was incubated at 25°C (Fig. 4B).

To optimize induction conditions, various concentrations of IPTG were used [46]. IPTG plays an important role in protein expression, but excessive IPTG is toxic to cell growth and reduces enzyme activity [47]. The OD_600_ reached 0.8 when the 18 h pre-culture medium was inoculated at a final concentration of 2% v/v and incubated for 2 h at 37°C. At this time, IPTG in different concentrations (0, 0.01, 0.025, 0.05, 0.1, 0.25, 0.5, and 1 mM) was added, and the cultures were further incubated for 48 h at 25°C in a shaking incubator. When IPTG was not added, bioindigo was produced in small quantities. Bioindigo production was the highest at 0.01 mM IPTG induction (Fig. 4C). As the concentration of IPTG increased, the bioindigo production decreased, and the induction with 1 mM IPTG yielded 2.3 times lower bioindigo than with 0.01 mM.

Tryptophan is converted to indole by TnaA, and indole is converted to indoxyl by FMO [48]. Indigo is synthesized by the non-enzymatic dimerization of indoxyl. Dimerization is a reaction that does not require enzymes and occurs naturally in the presence of oxygen. To optimize the amount of available oxygen for the maximum production of bioindigo, the shaking speed (rpm) of the incubator was adjusted to control the amount of dissolved oxygen at the test-tube scale. The shaking speed was set to 150, 200, and 250 rpm. Bioindigo production was the highest at 200 rpm, and production was reduced at low or high speeds (Fig. 4D).

Each enzyme has an optimal pH. When the pH is outside the optimal range, the activity of the enzyme slows down, and excessive pH denatures the enzyme [49]. The pH of the culture medium for bioindigo production was adjusted to different levels (5, 6, 7, 8, and 9). The bioindigo production at various pH ranges was tested. The control was kept at pH 7.23; the bioindigo production was quantified after 48 h of incubation (Fig. 4E). Bioindigo production was 5.33 mM at pH 8, 1.04 times higher than the control (5.12 mM). Bioindigo production decreased rapidly when the pH was higher or lower than the optimal pH range of 6–8.

### Effect of Tryptophan Concentration on Indigo Production and Time-Dependent Monitoring of the Reaction

**Fig. 5:**
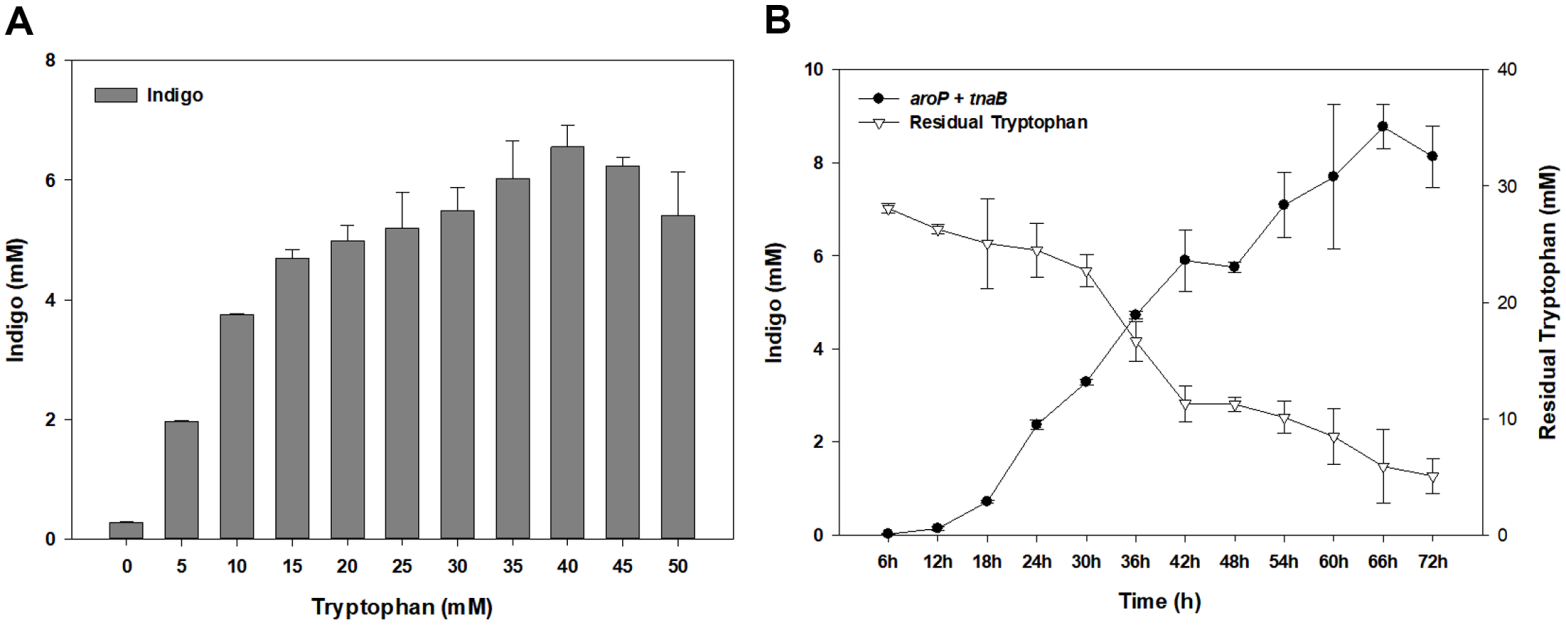
Production of indigo under optimal conditions. (**A**) Indigo production by tryptophan concentration. (**B**) Monitoring time-dependent production of indigo in recombinant strains.

To determine the appropriate concentration of tryptophan, 0, 5, 10, 15, 20, 25, 30, 35, 40, 45, and 50 mM of tryptophan were added to the TB medium and used as a culture medium for the *aroP*+*tnaB* strain. The culture was performed under the optimal conditions determined from the previous experiments (TB medium, 25°C, 0.01 mM IPTG induction, 200 rpm, pH 8). Bioindigo production increased rapidly as tryptophan concentration increased from 0 to 40 mM (Fig. 5A). The production was the highest at 6.55 mM when the concentration of tryptophan was 40 mM. When the substrate was used at concentrations greater than 40 mM, indigo production was reduced. This was assumed to be due to high tryptophan concentration, resulting in substrate inhibition. Therefore, 40 mM tryptophan was selected as the optimal substrate concentration for bioindigo production. It has been reported that indirubin, a structural isomer of indigo, is produced under several medium conditions in bioindigo production. Cysteine, which has a positive effect on indirubin production, was added at different concentrations to compare the production of indigo and indirubin. As the concentration of cysteine increased, the production of indirubin increased, whereas the production of indigo significantly decreased (Fig. S1).

Indigo production and residual tryptophan over time of the *aroP*+*tnaB* strain were monitored. The culture was performed with 40 mM tryptophan under optimal conditions. The cells were incubated for 72 h and harvested every 6 h to quantify the bioindigo and residual tryptophan. Bioindigo production gradually increased over time and was the highest at 8.77 mM (2.3 g/l) after 66 h of incubation (Fig. 5B). This is the highest production of bioindigo by microorganisms. The amount of residual tryptophan in the supernatant gradually decreased over time. It can be inferred from these results that the introduction of the tryptophan transporter increased the transport of tryptophan, helping substrate supply and consumption to produce more bioindigo.

**Fig. 6:**
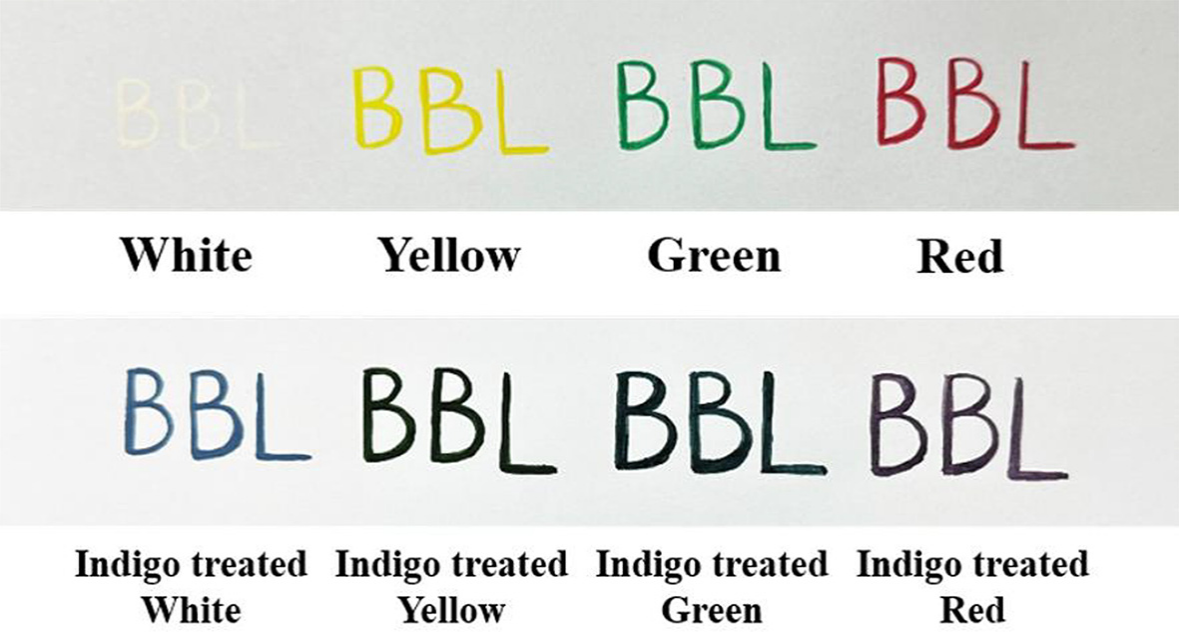
Application of watercolor dyeing method in bioindigo.

Bioindigo is not soluble in water and some of it may stick to the membrane, so loss may occur during the extraction process and the recovery yield may be low [50]. To obtain directly applicable bioindigo products with high production yield, the bioindigo-producing cells were harvested and destroyed by adding 1% SDS to the cell culture and heating it to obtain bioindigo inside the cells. The obtained bioindigo dye was recovered in powder form [19]. The recovered bioindigo was then applied as a dye to watercolor. A small amount of bioindigo powder was added to white, yellow, green, and red water and dissolved. Similar to other blue dyes, indigo-treated water colors mixed well with other colors and changed from white to blue, yellow to green, green to dark green, and red to purple (Fig. 6). This suggests that bioindigo can be used in color combinations similar to those of other blue dyes. The color was retained for 20 days. Although many studies have been conducted on single dyeing using indigo, they did not show miscibility and color retention with other colors, and this result showed significant compatibility of bioindigo with other colors as a source of blue color.

## Conclusion

Because microbial bioindigo production avoids many environmental and harmful issues associated with extraction and chemical synthesis, this method holds much promise once the production level reaches a large scale. To increase the production of bioindigo, we applied whole-cell systems with *FMO* and *tnaA* genes. We increased the uptake of tryptophan in *E. coli* by introducing the tryptophan transporter *aroP* and *tnaB* genes and established an indigo production system through the co-expression with *FMO* and *tnaA* genes. The introduction of a transporter increased the transport of tryptophan into the cell, which led to an increase in the production of the intermediate compound indole and the bioindigo product. After optimizing the culture conditions, we produced a maximum of 8.77 mM (2.3 g/l) indigo. This is the highest indigo yield from *E. coli* ever achieved (Table 1). In addition, by simply boiling the cells with SDS, bioindigo was recovered in powder form and immediately blended with other colors, as solvent-based recovery is very difficult because indigo is not well solubilized. Bioindigo was well mixed with other colors and the color was retained for a relatively long time, which was quite different from the bioindigo recovered by the solvent. Overall, this study highlights that the introduction of transporters and the application of combinatorial transporters are effective in improving the production of bioindigo.

## Supplemental Materials

Supplementary data for this paper are available on-line only at http://jmb.or.kr.



## Figures and Tables

**Table 1:** Bioindigo production through various microorganisms in previous studies.

Strain type	Substrate	Main enzyme	Titer	Remarks	References
*Pseudomonas putida*	Indole	Styrene monooxygenase (StyAB) Indole monooxygenase	52.13 mg/L	Overexpression of StyAB enhanced indole monooxygenase activity and led to seven-fold higher indigo yield in the recombinant strain.	(Cheng *et al*., 2016)
*Acinetobacter* sp. ST-550	Indole	-	0.3 mg/L	Indigo production increased in the presence of a large volume of diphenylmethane and a high level of indole.	(Doukyu *et al*., 2002)
*Acinetobacter* sp. PP-2	Indole and phenol	-	202.9 mg/L	Response surface methodology was applied to optimize the concentration of substrate phenol and indole.	(Qu *et al*., 2010)
*Comamonas* sp. MQ	Indole and naphthalene	Naphthalene dioxygenase	32.2 mg/L	First study of indigo biosynthesis using *Comamonas* sp.	(Qu *et al*., 2012b)
Recombinant *E. coli*	Tryptophan	Flavin-containing monooxygenase (FMO)	920 mg/L	Upstream sequence size of *FMO* gene was optimized and response surface methodology used	(Han *et al*., 2008)
Recombinant *E. coli*	Tryptophan	FMO Cyclopropane-fatty acid-acyl-phospholipid synthase (Cfa)	1.08 g/L	Changes in the cell membrane phospholipid composition affect indigo production	(Ham *et al*., 2023)
Recombinant *E. coli*	Tryptophan	Aromatic amino acid transport protein AroP, Low affinity tryptophan permease (TnaB)	2.30 g/L	Transporter gene expression in *E. coli* increases uptake of L-tryptophan, affecting indigo production	This study

**Table 2:** Bacterial strains and plasmids used in this study.

Strain and plasmid	Genotype and strain description	Source or reference
Bacterial strains		
*Escherichia coli* DH5α	F^-^ *ϕ 80lacZ M15 endA recA hsdR(rk^-^mk^-^) supE thi gyrA relAΔ(lacZYA-argF)U169*	Taylor *et al*
*E. coli* BL21 (DE3)	F^-^ *ompT hsdS_B_ (r_B_^-^m_B_^-^) gal dcm*	Novagen
*E. coli* BL21 *FMO*+*tnaA*	*E. coli* BL21 Star (DE3) harboring pCDF duet-1::*FMO*::*tnaA*, Spec^r^	In this study
*E. coli* BL21 *aroP*	*E. coli* BL21 Star (DE3) harboring pCDF duet-1::*FMO*::*tnaA* and pET 24ma::*aroP*, Spec^r^/Km^r^	In this study
*E. coli* BL21 *tnaB*	*E. coli* BL21 Star (DE3) harboring pCDF duet-1::*FMO*::*tnaA* and pET 24ma::*tnaB*, Spec^r^/Km^r^	In this study
*E. coli* BL21 *Mtr*	*E. coli* BL21 Star (DE3) harboring pCDF duet-1::*FMO*::*tnaA* and pET 24ma::*Mtr*, Spec^r^/Km^r^	In this study
*E. coli* BL21 *aroP*+*tnaB*	*E. coli* BL21 Star (DE3) harboring pCDF duet-1::*FMO*::*tnaA* and pET 24ma::*aroP*::*tnaB*, Spec^r^/Km^r^	In this study
*E. coli* BL21 *aroP*+*Mtr*	*E. coli* BL21 Star (DE3) harboring pCDF duet-1::*FMO*::*tnaA* and pET 24ma::*aroP*::*Mtr*, Spec^r^/Km^r^	In this study
Plasmids		
pCDF duet-1	Spec^r^. 2 MCS site with T7 promoter, lac operator, RBS. CloDF13 replicon.	Novagen
pET 24ma	Km^r^. 1 MCS site with T7 promoter, lac operator, RBS. p15A replicon.	Novagen
pCDF duet-1::*FMO*	*FMO* gene of *Methylophaga aminisulfidivorans* inserted into pCDF duet‐1	In this study
pCDF duet-1::*FMO*::*tnaA*	*tnaA* gene of *Escherichia coli* K12 inserted into pCDF duet‐1::*FMO*	In this study
pET 24ma::*aroP*	*aroP* gene of *Escherichia coli* K12 inserted into pET 24ma	In this study
pET 24ma::*tnaB*	*tnaB* gene of *Escherichia coli* K12 inserted into pET 24ma	In this study
pET 24ma::*Mtr*	*Mtr* gene of *Escherichia coli* K12 inserted into pET 24ma	In this study
pET 24ma::*aroP*::*tnaB*	*tnaB* gene of *Escherichia coli* K12 inserted into pET 24ma::*aroP*	In this study
pET 24ma::*aroP*::*Mtr*	*Mtr* gene of *Escherichia coli* K12 inserted into pET 24ma::*aroP*	In this study
