# Supplementary figures and images for: Tryptophan-Based Hyperproduction of Bioindigo by Combinatorial Overexpression of Two Different Tryptophan Transporters
Source: J Microbiol Biotechnol. 2023 Nov 30;34(4):969–77. doi: 10.4014/jmb.2308.08039 (PMC11091664; doi:10.4014/jmb.2308.08039)

**Supplementary Fig. S1.**

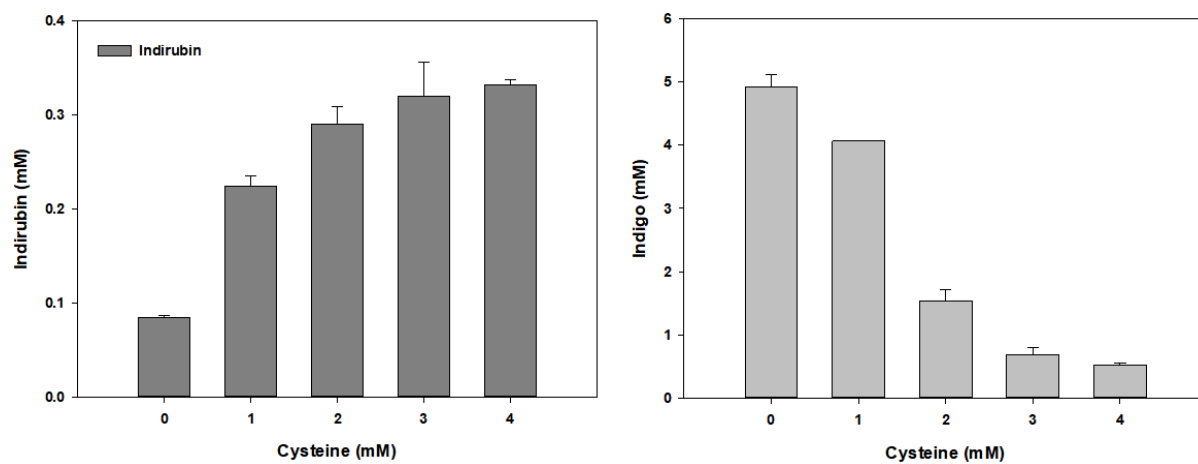

Supplement: Supplementary file 1 [file jmb-34-4-969-supple.pdf]
